# Supplementary material for: Wildlife Dermatophytoses in Central Italy (Umbria and Marche Regions): A Fifteen-Year Investigation (2010–2024)
Source: J Fungi (Basel). 2025 Oct 21;11(10):753. doi: 10.3390/jof11100753 (PMC12565737; doi:10.3390/jof11100753)
Supplement: Supplementary file 1 [file jof-11-00753-s001.zip › jof-3861117-supplementary.pdf]

Sample ID	Collection date	Location (region)	Host species	End-point PCR (ITS) + Sanger sequencing results	***Trichophyton mentagrophytes* genotype**
1	2010	Marche	Red fox	*T. mentagrophytes* complex	/
2	2010	Marche	Red fox	*M. canis*	/
3	2010	Marche	Red fox	*T. mentagrophytes* complex	/
4	2010	Marche	Red fox	*M. canis*	/
5	2010	Marche	Red fox	*M. canis*	/
6	2010	Marche	Red fox	*M. canis*	/
7	2012	Umbria	Grey squirrel	*T. mentagrophytes* var. *mentagrophytes*	III*
8	2012	Umbria	Grey squirrel	*P. mirabile*	/
9	2012	Umbria	Red squirrel	*T. mentagrophytes* var. *interdigitale*	II*
10	2012	Umbria	Red squirrel	*T. mentagrophytes* var. *interdigitale*	II*
11	2012	Umbria	Red squirrel	*T. mentagrophytes* var. *interdigitale*	II*
12	2012	Umbria	Grey squirrel	*P. mirabile*	/
13	2012	Umbria	Red squirrel	*T. mentagrophytes* var. *interdigitale* + *M. canis*	II*
14	2012	Umbria	Grey squirrel	*P. mirabile*	/
15	2013	Umbria	Grey squirrel	*T. mentagrophytes* complex	/
16	2015	Umbria	Grey squirrel	*T. mentagrophytes* var. *interdigitale*	II*
17	2016	Umbria	Grey squirrel	*T. mentagrophytes* var. *interdigitale*	II*
18	2016	Umbria	Grey squirrel	*T. mentagrophytes* complex	/
19	2016	Umbria	Grey squirrel	*P. mirabile*	/
20	2016	Umbria	Grey squirrel	*P. mirabile*	/
21	2016	Umbria	Grey squirrel	*P. mirabile*	/
22	2016	Umbria	Grey squirrel	*P. mirabile*	/
23	2016	Umbria	Grey squirrel	*A. uncinatum*	/
24	2016	Umbria	Grey squirrel	*P. mirabile*	/
25	2016	Umbria	Grey squirrel	*P. mirabile*	/
26	2016	Umbria	Grey squirrel	*A. thuringiense*	/
27	2016	Umbria	Grey squirrel	*P. mirabile*	/
28	2016	Umbria	Grey squirrel	*T. mentagrophytes* complex	/
29	2016	Umbria	Grey squirrel	*P. mirabile*	/
30	2016	Umbria	Grey squirrel	*P. mirabile*	/
31	2016	Umbria	Grey squirrel	*A. thuringiense*	/
32	2016	Umbria	Grey squirrel	*P. mirabile*	/
33	2016	Umbria	Grey squirrel	*P. mirabile*	/
34	2016	Umbria	Grey squirrel	*P. mirabile*	/
35	2016	Umbria	Grey squirrel	*A. currey*	/
36	2016	Umbria	Grey squirrel	*P. mirabile*	/
37	2016	Umbria	Grey squirrel	*A. uncinatum*	/
38	2016	Umbria	Grey squirrel	*T. mentagrophytes* complex	/
39	2016	Umbria	Grey squirrel	*P. mirabile*	/
40	2016	Umbria	Grey squirrel	*P. mirabile*	/
41	2016	Umbria	Grey squirrel	*P. mirabile*	/
42	2016	Umbria	Grey squirrel	*A. uncinatum*	/
43	2016	Umbria	Grey squirrel	*A. currey*	/
44	2016	Umbria	Grey squirrel	*P. mirabile*	/
45	2016	Umbria	Grey squirrel	*A. currey*	/
46	2017	Umbria	Grey squirrel	*T. mentagrophytes* var. *interdigitale*	II*
47	2017	Umbria	Grey squirrel	*T. mentagrophytes* var. *interdigitale*	II*
48	2017	Umbria	Grey squirrel	*A. thuringiense*	/
49	2017	Umbria	Grey squirrel	*P. mirabile*	/
50	2017	Umbria	Grey squirrel	*P. mirabile*	/

51	2017	Umbria	Grey squirrel	*P. mirabile*	/
52	2017	Umbria	Grey squirrel	*P. mirabile*	/
53	2017	Umbria	Grey squirrel	*P. mirabile*	/
54	2017	Umbria	Grey squirrel	*P. mirabile*	/
55	2017	Umbria	Grey squirrel	*P. mirabile*	/
56	2017	Umbria	Grey squirrel	*P. mirabile*	/
57	2017	Umbria	Grey squirrel	*P. mirabile*	/
58	2017	Umbria	Grey squirrel	*P. mirabile*	/
59	2017	Umbria	Grey squirrel	*P. mirabile*	/
60	2017	Umbria	Grey squirrel	*P. mirabile*	/
61	2017	Umbria	Grey squirrel	*P. mirabile*	/
62	2017	Umbria	Grey squirrel	*P. mirabile*	/
63	2017	Umbria	Grey squirrel	*P. mirabile*	/
64	2017	Umbria	Grey squirrel	*P. mirabile*	/
65	2017	Umbria	Grey squirrel	*P. mirabile*	/
66	2018	Umbria	Grey squirrel	*A. thuringiense*	/
67	2018	Umbria	Grey squirrel	*P. mirabile*	/
68	2018	Umbria	Grey squirrel	*P. mirabile*	/
69	2021	Umbria	European hedgehog	*P. mirabile*	/
70	2021	Umbria	Red squirrel	*T. mentagrophytes* var. *mentagrophytes*	III*
71	2021	Umbria	Red squirrel	*T. mentagrophytes* var. *interdigitale*	II*
72	2021	Umbria	Grey squirrel	*P. mirabile*	/
73	2021	Umbria	Grey squirrel	*P. mirabile*	/
74	2022	Umbria	Red fox	*A. eboreum*	/
75	2023	Umbria	European badger	*A. eboreum*	/
76	2023	Umbria	Red fox	*A. currey*	/
77	2024	Umbria	Crested porcupine	*A. eboreum*	/

Table S1. Detailed information about the 77 positive samples.
